# The Impact of Longitudinal Substance Use Patterns on the Risk of Opioid Agonist Therapy Discontinuation: A Repeated Measures Latent Class Analysis

**DOI:** 10.1007/s11469-023-01098-8

**Published:** 2023-06-28

**Authors:** Zishan Cui, Mohammad Karamouzian, Michael Law, Kanna Hayashi, M-J Milloy, Thomas Kerr

**Affiliations:** 1 British Columbia Centre on Substance Use, 400-1045 Howe Street, Vancouver, BC Canada; 2 School of Population and Public Health, University of British Columbia, 2206 E Mall, Vancouver, BC Canada; 3 Centre on Drug Policy Evaluation, Saint Michael’s Hospital, 36 Queen St E, Toronto, ON Canada; 4 Centre for Health Services and Policy Research, University of British Columbia, 2206 E Mall, Vancouver, BC Canada; 5 Faculty of Health Sciences, Simon Fraser University, 8888 University Drive, Burnaby, BC Canada; 6 Department of Medicine, University of British Columbia, 317-2194 Health Sciences Mall, Vancouver, BC Canada

**Keywords:** Opioid Agonist Therapy (OAT), OAT Discontinuation, OAT Retention, Polysubstance Use, Repeated Measures Latent Class Analysis (RMLCA), Marginal Structural Modeling

## Abstract

Polysubstance use is prevalent among individuals on opioid agonist treatment (OAT), yet past studies have focused primarily on distinct substances and their association with OAT retention. Data was collected from two prospective cohorts between 2005 and 2020 in Vancouver, Canada. Among 13,596 visits contributed by 1445 participants receiving OAT, we employed repeated measures latent class analysis using seven indicators and identified four longitudinal substance use classes. Using marginal structural Cox modeling, we found that compared to the primarily crack use class, the two opioid and stimulant use classes carried a higher risk of OAT discontinuation, while the primarily cannabis and crack use class had a lower OAT discontinuation risk. Our findings highlight the need for integrated treatment strategies to manage the co-use of opioids and stimulants during receipt of OAT and suggest future research should explore the potential of cannabis as a harm reduction strategy or adjunctive treatment to OAT.

Word count: 150/150.

**Supplementary Information:**

The online version contains supplementary material available at 10.1007/s11469-023-01098-8.

## Introduction

The rate of opioid-related overdose continues to increase in the United States (US) and Canada (Belzak & Halverson, 2018; Kariisa et al., 2019), largely driven by synthetic fentanyl and its analogues in the illicit drug supply (Government of Canada, 2022a; NIDA, 2021; The Lancet Public Health, 2018). British Columbia (BC), Canada, has consistently reported the highest opioid-related deaths per capita in Canada (Government of Canada 2022b). Following a significant increase in opioid-related overdose deaths, the province declared a public health emergency in 2016 (BC Centre for Disease Control, 2017). Since then, BC Coroners Service has reported over 9,000 illicit drug toxicity deaths and the death rate has doubled from 20.4 to 100,000 population in 2016 to 43.6 in 2021 (BC Coroners Service, 2020). Multiple policy responses, including the scale-up of opioid agonist therapy (OAT) and harm reduction programs, have been deployed (MacDougall et al., 2019).

OAT is the first-line treatment for opioid use disorder (OUD) (Bahji et al., 2019; Sordo et al., 2017). Various interventional and observational studies have demonstrated the effectiveness of different modalities of OAT in reducing overdose, morbidity, and all-cause mortality among people living with OUD (Bahji et al., 2019; Sordo et al., 2017). In Canada, methadone was approved in the 1960s and buprenorphine/naloxone was approved in 2007 to treat OUD (Priest et al., 2019). The national clinical practice guidelines for the management of OUD recommend buprenorphine/naloxone as a first-line treatment, progressing toward methadone if required; then, if needed and appropriate, toward slow-release oral morphine and injectable forms of OAT (Bruneau et al., 2018; Lu, 2020). Alongside clinical benefits, retention on OAT is also associated with decreased criminal activity and improved occupational, social and psychological functioning, resulting in total health and social savings exceeding twelve-fold compared to dollars invested in the OAT treatment itself (National Institute on Drug Abuse, 2012).

Despite these benefits, treatment discontinuation remains a major challenge for OAT programs (Banta-Green et al., 2009; Kennedy et al., 2022; O’Connor et al., 2020; Timko et al., 2016). While a minimum of one-year retention in OAT is recommended to achieve the best outcomes (Gibson et al., 2008; Zhang et al., 2003; Zhu et al., 2018), in a systematic review published in 2020, O’Connor et al. summarized 67 studies and showed that the median OAT retention rates among these studies at one year since treatment initiation was only 60.7% (O’Connor et al., 2020). Factors associated with reduced OAT retention included criminal activity involvement or incarceration, patients’ negative attitudes towards OAT, as well as ongoing unregulated drug use, cocaine and heroin use in particular (O’Connor et al., 2020).

Previous studies have primarily focused on specific types of substances and their associations with OAT retention. Unregulated drug use including heroin use and cocaine use were most frequently found to be associated with reduced OAT retention (Banta-Green et al., 2009; Deck & Carlson, 2005; Montalvo et al., 2019; Peles et al., 2008, 2018; Proctor et al., 2015; Sullivan et al., 2010; Wan Shakira et al., 2017; Williamson et al., 2006), while the association between methamphetamine use and reduced OAT retention was found to be significant in some settings but not others (Banta-Green et al., 2009; Deck & Carlson, 2005; Mackay et al., 2021; Peles et al., 2008; Proctor et al., 2015). Moreover, alcohol consumption was generally found to be not associated with OAT retention (Amiri et al., 2018; Kayman et al., 2006; Kelly et al., 2011), while the association between cannabis use and OAT retention remains unclear due to the conflicting results produced from observational studies (Franklyn et al., 2017; Hser et al., 2014; Hurd et al., 2015; Scavone et al., 2013; Socías et al., 2018; Weizman et al., 2004; Zielinski et al., 2016). The existing evidence underscores the complexity of understanding the relationship between different substance use patterns and OAT retention.

In recent years, rates of polysubstance use are rising among individuals with OUD (Cicero et al., 2020; Crummy et al., 2020; Hassan & Le Foll, 2019; Karamouzian et al., 2022b) and this has posed additional challenges for long-term retention in OAT (O’Connor et al., 2020; Wang et al., 2017). For individuals engaged in polysubstance use, OAT only addresses a portion of their treatment needs and could be less effective with regard to reducing harms associated with unregulated opioid use (Senbanjo et al., 2009). Nonetheless, analyses focused on polysubstance use have typically been oversimplified via a focus on the use of more than two types of drugs or any concurrent drug use (Banks et al., 2019; Hjemsæter et al., 2019; Jongenelis et al., 2019). These definitions fail to distinguish distinct substance use patterns according to different combinations of substance co-use. Given the challenges associated with measuring polysubstance use in practice due to potential variations of all possible substance use combinations, the impact of the longitudinal patterns of substance use on OAT retention has not been fully characterized. Therefore, our study aimed to characterize longitudinal substance use patterns among people receiving OAT and evaluate the association between different substance use patterns and the risk of OAT discontinuation. Such understanding is clinically important to inform tailored drug treatment strategies such as integrated treatment plans, and thus help improve OAT retention and associated outcomes.

## Methods

### Study Sample

This study was conducted using two ongoing prospective cohorts: the Vancouver Injection Drug Users Study (VIDUS) and AIDS Care Cohort to Evaluate Exposure to Survival Services (ACCESS) study. These cohorts have been described previously (Kerr et al., 2005; Wood et al., 2004). In brief, participants of VIDUS and ACCESS have been recruited through self-referral, word of mouth, and street outreach primarily from the Downtown Eastside neighbourhood of Vancouver, which is characterized by high rates of structural marginalization, including criminalization, among people who use drugs (Friedel & Staak, 1993). VIDUS enrolls HIV-seronegative adults who self-report having injected unregulated drugs in the month prior to enrolment and ACCESS enrolls people who are living with HIV and have used at least one drug (other than or in addition to cannabis) in the month prior to enrolment. Once enrolled, at baseline and semi-annually thereafter, participants complete an interviewer-administered questionnaire obtaining information concerning socio-demographic characteristics, drug use patterns, risk behaviours, as well as health care utilization. The study instruments and follow-up procedures for each study are harmonized to allow for combined analyses. VIDUS participants who HIV seroconvert following recruitment would be transferred into the ACCESS study. Participants receive a $40 (CDN) honorarium for each study visit. All eligible participants provided written informed consent. The studies have been approved by the University of British Columbia/Providence Health Care Research Ethics Board.

Between December 2005 and March 2020, 2416 participants were recruited from VIDUS and ACCESS. We further restricted our study sample to those participants who: (1) self-reported receiving OAT during the past six months prior to their visit during the study period; and (2) completed at least one follow-up visit within one year thereafter. OAT included methadone, buprenorphine-naloxone, slow-release oral morphine, or injectable OAT. Participants who discontinued OAT and then restarted on OAT at a subsequent follow-up visit were considered at risk for discontinuation again from that subsequent follow-up visit and were included in the analyses. After applying sample restriction, a total of 1445 participants were included in our study. Of these, 908 (62.8%) participants were from VIDUS (i.e., HIV-seronegative) and 537 (37.2%) were from ACCESS (i.e., HIV-seropositive).

### Study Variables

The primary outcome was time to OAT discontinuation, defined as reporting having not received OAT during the previous six months prior to the interview date of a follow-up visit during the study period. The date of discontinuation was estimated as the midpoint between the last visit in which receiving OAT was reported and the first visit in which not receiving OAT was reported (Mackay et al., 2021). Additionally, as the majority of our participants receiving OAT were on methadone, we conducted a sensitivity analysis restricting the events to methadone discontinuation, defined as OAT discontinuation among participants receiving methadone. Participants who switched from methadone to other types of OAT were considered as censoring rather than methadone discontinuation in the sensitivity analysis (Mackay et al., 2021).

The main exposure variable was longitudinal substance use class, constructed using repeated measures latent class analysis (RMLCA) with seven binary substance use indicators including regular (i.e., at least weekly) unregulated opioid use (heroin or fentanyl), regular non-medical prescription opioid use, regular methamphetamine use, regular cocaine use, regular crack use, regular cannabis use, and heavy alcohol use (defined as > 3 standard drinks per occasion, or > 7 drinks per week for females; > 4 drinks per occasion, or > 14 drinks per week for males) (NIAAA, 2016). Our substance use variables, aside from alcohol use, were originally collected through multiple-choice questions. Participants were presented with a list of drugs and asked to indicate the frequency of use for each drug, including options such as daily, weekly, monthly, any use, or no use in the last six months. All substance use indicators corresponded to the past six months prior to the interview date. RMLCA is an extension of the latent class analysis to analyze longitudinal data structures, which allows for flexibility in identifying distinct patterns of substance use across different timepoints (Collins LM and Lanza ST 2010; Patrick et al., 2020). Given it is challenging to identify clinically meaningful latent classes due to the large number of possible combinations using the original categorical variables, we opted to dichotomize our indicators to reflect at-least-weekly use considering clinical relevance, existing research, and methodological guidance (Karamouzian, Buxton et al., 2022; Karamouzian, Pilarinos, Karamouzian et al., 2022a, b; Sinha et al., 2020; Weller et al., 2020). To select the number of latent classes, two to seven-class RMLCA model were assessed and five model fit indices were reviewed (i.e., Akaike Information Criterion [AIC], Corrected AIC, Bayesian Information Criterion [BIC], adjusted BIC, and entropy) (Nylund et al., 2007). The final solution was determined considering a combination of the model fit indices and interpretability and utility of the final model in light of existing knowledge (Nylund et al., 2007; Vermunt, 2010). We then assigned the substance use class corresponding to the highest posterior membership probability produced by RMLCA to each participants’ study visit (Vermunt, 2010). Additionally, to ensure the robustness of the identified patterns, we conducted a sensitivity analysis using daily drug use as indicators.

The selection of potential confounders of the association between substance use class and OAT discontinuation was informed by existing research (Banks et al., 2019; Banta-Green et al., 2009; Gibson et al., 2008; Jongenelis et al., 2019; Kennedy et al., 2022; O’Connor et al., 2020; Senbanjo et al., 2009; Timko et al., 2016; Wang et al., 2017; Zhang et al., 2003; Zhu et al., 2018). Potential sociodemographic confounders included: age; self-identified gender (male vs. female/transgender/two-spirit); self-reported ethnicity/ancestry (White vs. Indigenous vs. Black or other person of color); and cohort designation (VIDUS vs. ACCESS). Potential social-structural confounders included living in unstable housing (hotel/shelter/recovery house/street/other vs. apartment/house); residence in the Downtown Eastside; sex work involvement; incarceration; drug-dealing involvement; and difficulty accessing addiction treatment. We also controlled for new initiates of OAT as we hypothesized they would be less likely to stabilize on OAT and thus would have higher risk of discontinuation. New initiates of OAT were defined as a participant reporting not being on OAT in an immediately preceding study visit. All variables except for ethnicity and education were time-varying variables. All time-varying variables referred to experiences in the past six months prior to the study visit. We used these time-varying variables collected on one visit immediately preceding an OAT discontinuation outcome to exclude the possibility that they are consequences rather than predictors of OAT discontinuation.

### Statistical Analysis

First, we compared the baseline sample characteristics stratified by OAT discontinuation at any point during the study period, using the Pearson’s *χ*^2^ test for categorical variables and Wilcoxon Rank Sum test for continuous variables. We also estimated the incidence rates alongside 95% confidence intervals (CI) of OAT discontinuation for the entire sample as well as stratified by substance use class.

We fit univariable extended Cox regression to estimate the unadjusted association between each covariate and time to OAT discontinuation. Next, we used a marginal structural Cox model to estimate the association between different substance use classes and time to OAT discontinuation (Hernán et al., 2000; Jensen et al., 2016; Suarez et al., 2008). This method can account for time-varying variables that are simultaneously confounders and affected by previous substance use class exposure (e.g., living in unstable housing, drug dealing involvement) and can also adjust for potential differential lost to follow up. Loss to follow up was defined as the last interview date being longer than 2 years before the study end date (i.e., March 16, 2020). We calculated stabilized weights which combine information on the probability of a person being assigned in each substance use class and censoring history given the baseline and time-updated covariates (Hernán et al., 2000). The number of visits containing missing data was minimal (< 1% for each variable), and therefore visits containing missing covariates were removed from the marginal structural Cox model. Sensitivity analyses were also conducted to model time to methadone discontinuation. All statistical tests were two-sided and considered statistically significant at *p* < 0.05. All analyses were conducted using SAS version 9.4 (SAS Institute, Cary, North Carolina, United States).

## Results

Between December 2005 and March 2020, 1445 participants included in our study were followed for a median of 3.8 years (1st to 3rd quartile [Q1-Q3] = 1.3-7.7) and contributed a median of 7 visits (Q1-Q3 = 3-15), resulting in a total of 13,596 visits; 94.0% of these visits were on methadone. Of all study visits, nearly half (48.4%) involved regular use of at least two types of drugs, 35.2% involved regular opioid use, 60.0% involved regular stimulant use, and 25.6% involved regular use of both opioids and stimulants. In total, 121 combinations of regular drug use were reported. The baseline characteristics of all participants stratified by OAT discontinuation during study follow-up are presented in Table 1. At baseline, the median age of the participants was 42 (Q1-Q3: 35-49); 847 (58.6%) self-identified as being male; 886 (61.9%) self-identified as White, and 492 (34.4%) self-identified as being of Indigenous ancestry.

**Table 1:** Baseline sample characteristics stratified by OAT discontinuation during follow-up among participants receiving OAT in Vancouver, Canada (N = 1445)

			OAT discontinuation		
	Total	Never		Ever	
	(N = 1445)	(N = 727)		(N = 718)	
Variables	N (%)	N (%)		N (%)	*p*-value
Substance use class^a^					
Primarily crack use	464 (32.1%)	238 (32.7%)		226 (31.5%)	0.763
Primarily cannabis & crack use	214 (14.8%)	111 (15.3%)		103 (14.4%)	
Primarily opioid & crack/cocaine use	529 (36.6%)	265 (36.5%)		264 (36.8%)	
Primarily opioid & methamphetamine use	238 (16.5%)	113 (15.5%)		125 (17.4%)	
Age (median (Q1-Q3))	42 (35–49)	43 (36–49)		41 (34–48)	0.007
Male gender	847 (58.6%)	434 (59.7%)		413 (57.5%)	0.401
Ethnicity/Ancestry					
White	886 (61.9%)	472 (65.4%)		414 (58.3%)	0.018
Indigenous	492 (34.4%)	223 (30.9%)		269 (37.9%)	
Black and other person of color	54 (3.8%)	27 (3.7%)		27 (3.8%)	
Cohort					
ACCESS	537 (37.2%)	308 (42.4%)		229 (31.9%)	< 0.001
VIDUS	908 (62.8%)	419 (57.6%)		489 (68.1%)	
Unstable housing	1025 (71.2%)	511 (70.7%)		514 (71.7%)	0.672
Living in Downtown Eastside	961 (67.1%)	478 (66.2%)		483 (67.9%)	0.487
Sex work involvement^a^	256 (17.7%)	135 (18.6%)		121 (16.9%)	0.393
Incarceration^a^	218 (15.1%)	103 (14.2%)		115 (16.0%)	0.321
Drug-dealing^a^	473 (32.8%)	243 (33.5%)		230 (32.0%)	0.561
Unable to access addiction treatment^a^	89 (6.2%)	40 (5.5%)		49 (6.8%)	0.299
Newly OAT initiatives	474 (32.8%)	172 (23.7%)		302 (42.1%)	< 0.001

^a^Variables refers to the last six months prior to the interview date

**Fig. 1:**
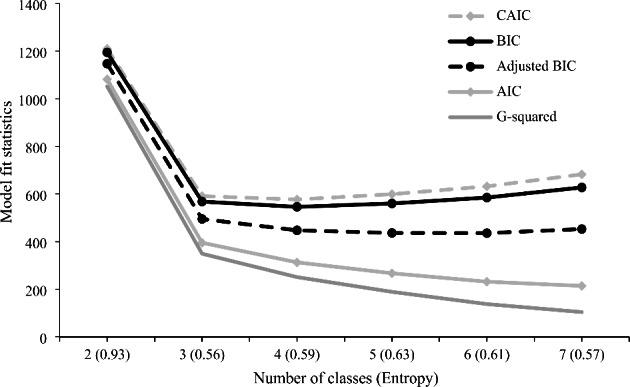
Repeated measures latent class model fit statistics

**Fig. 2:**
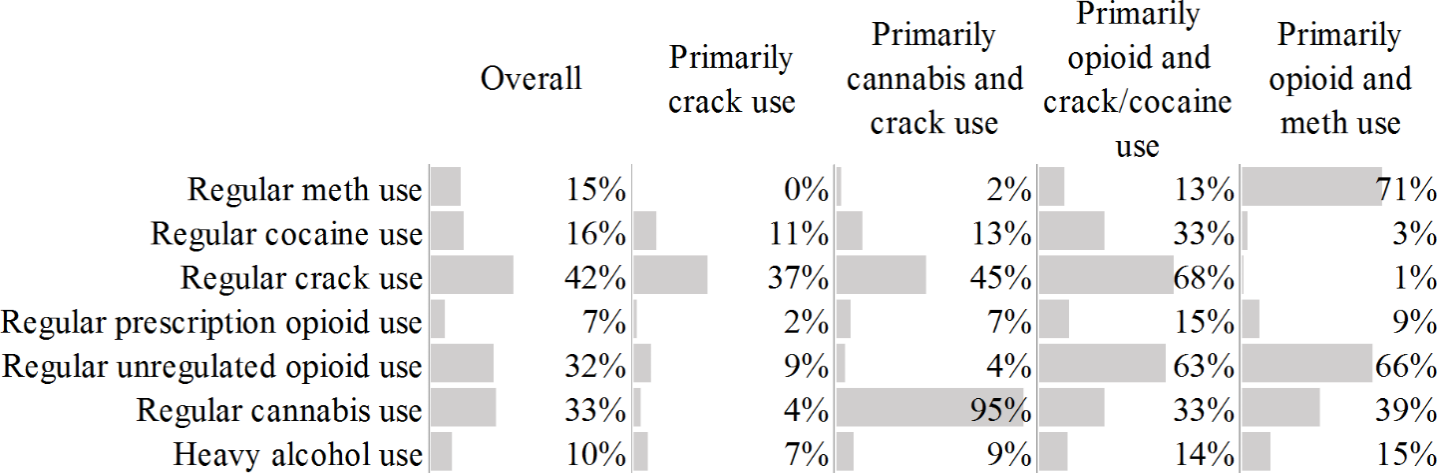
Four latent substance use class item response probability loadings on substance use indicators among participants receiving OAT in Vancouver, Canada. Regular: at least weekly. All substance use indicators refer to the last six months prior to the interview date

As shown in Fig. 1, two to seven-class RMLCA models were assessed. The four-class model was chosen due to its best interpretability, utility and parsimony of the classes suggested by the lowest BIC and CAIC value, and its corresponding item response probabilities loading on the substance use indicators are presented in Fig. 2. Of all visits, 6250 (46.0%) visits were identified as Class 1 (primarily crack use) and 2666 (19.6%) visits were identified as Class 2 (primarily cannabis and crack use). Participants in these two classes were unlikely to use unregulated opioids regularly, while nearly half (~ 40%) of the participants were likely to use crack regularly. Participants in class 2 were also likely to use cannabis regularly. Besides, 2817 (20.7%) visits were identified as Class 3 (primarily opioid and crack/cocaine use) and 1863 (13.7%) visits were identified as Class 4 (primarily opioid and methamphetamine use). Participants in these two classes were likely to use unregulated opioids regularly. Participants in class 3 were also likely to use cocaine/crack regularly, while participants in class 4 were also likely to use methamphetamine regularly. Our sensitivity analysis using daily drug use as indicators identified similar substance use patterns (Figure S1). The only difference being that the sensitivity analysis presented three classes instead of four, and it failed to distinguish the differences in cannabis use within the two classes characterized by moderate crack use in the absence of unregulated opioid use.

**Fig. 3:**
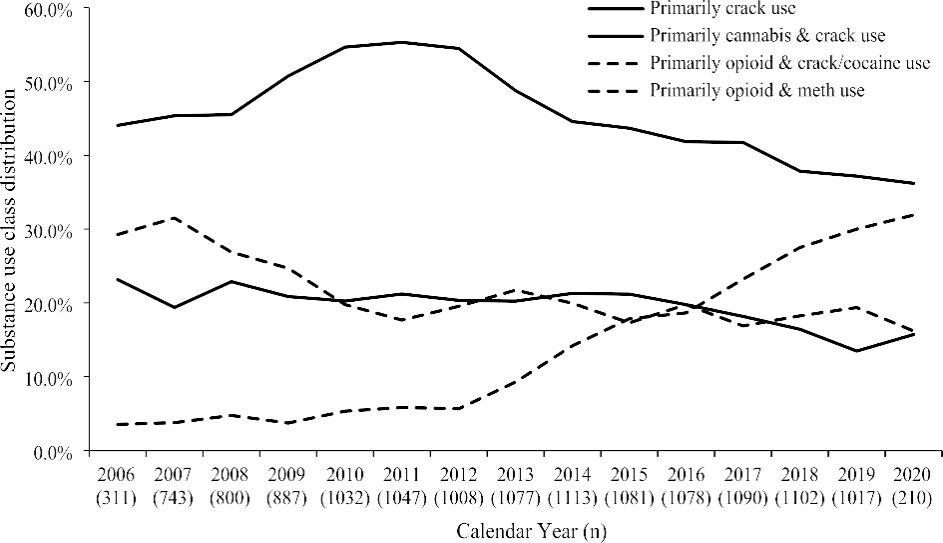
Substance use class distributions by calendar year among participants receiving OAT in Vancouver, Canada

**Fig. 4:**
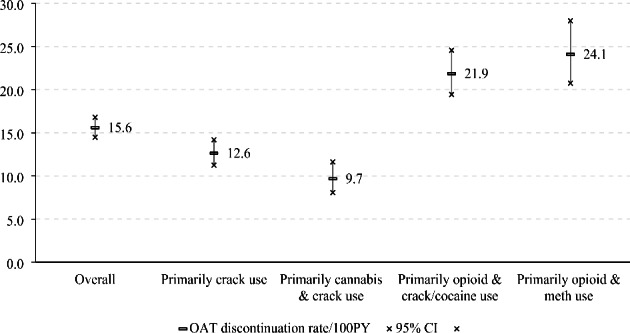
OAT discontinuation rate for the entire sample and stratified by substance use class among participants receiving OAT in Vancouver, Canada

Figure 3 shows the evolving substance use patterns among our participants receiving over the study period. From 2006 to 2019, the total prevalence of the two polysubstance use classes involving regular use of both opioid and stimulant increased from 32.8% to 49.4%. In particular, the prevalence of primarily opioid and methamphetamine use class increased from 3.5% to 2006 to 30.0% in 2019, while the prevalence of primarily opioid and crack/cocaine use class decreased from 29.3% to 2006 to 19.4% in 2019. During the follow-up, 718 (49.7%) participants discontinued OAT at least once. As shown in Fig. 4, the overall OAT discontinuation rate was 15.6 (95% CI = 14.5–16.8) per 100 person-years [PY]. Stratified by substance use class, the OAT discontinuation rates for the two polysubstance use classes (primarily opioid and crack/cocaine use class: 21.9 per 100 PY; primarily opioid and methamphetamine class 24.1 per 100 PY) were about twice as large as the rates for the primarily crack use (12.6 per 100PY) or the primarily cannabis and crack use class (9.7 per 100PY).

Table 2 presents the results of the unadjusted Cox models studying the associations between each covariate and time to OAT discontinuation. In Table 3, the marginal structural Cox model shows that compared to the primarily crack use class, the two polysubstance use classes involving regular use of both opioid and stimulant were associated with higher OAT discontinuation risk (primarily opioid and crack/cocaine use class: adjust hazard ratio [aHR] = 1.56, 95% CI = 1.28–1.90; primarily opioid and methamphetamine use class: aHR = 1.75, 95% CI = 1.39–2.20), and the primary cannabis and crack use class was associated with lower OAT discontinuation risk (aHR = 0.72, 95% CI = 0.57–0.92). Sensitivity analyses modeling time to methadone discontinuation showed similar results as in the primary models (Tables 2 and 3).

**Table 2:** Univariable extended Cox regression model for the effect of substance use classes on risk of OAT/methadone discontinuation among participants receiving OAT in Vancouver, Canada

	Univariable Extended Cox Model	
	HR (95% CI)	HR (95% CI)
	OAT discontinuation (n = 13,596)	Methadone discontinuation (n = 12,773)
Substance Use Class^a^		
Primarily crack use	Ref	Ref
Primarily cannabis & crack use	0.75 (0.60–0.94)	0.74 (0.58–0.94)
Primarily opioid & crack/cocaine use	1.77 (1.50–2.10)	1.84 (1.55–2.19)
Primarily opioid & meth use	1.93 (1.58–2.37)	1.84 (1.47–2.30)
Age (per year increase)	0.96 (0.95–0.97)	0.95 (0.95–0.96)
Male gender	0.92 (0.79–1.08)	0.87 (0.74–1.03)
Ethnicity/Ancestry		
White	Ref	Ref
Indigenous	1.36 (1.15–1.61)	1.37 (1.15–1.63)
Black and other person of color	1.31 (0.89–1.93)	1.43 (0.96–2.12)
Cohort		
ACCESS	Ref	Ref
VIDUS	1.34 (1.13–1.58)	1.38 (1.16–1.63)
Unstable housing	1.22 (1.05–1.42)	1.24 (1.06–1.45)
Living in Downtown Eastside	1.06 (0.92–1.23)	1.07 (0.91–1.25)
Sex Work^a^	1.37 (1.10–1.70)	1.44 (1.15–1.80)
Incarceration^a^	1.94 (1.58–2.38)	1.98 (1.59–2.47)
Drug-dealing^a^	1.34 (1.15–1.57)	1.37 (1.16–1.61)
Unable to access addiction treatment^**a**^	1.63 (1.25–2.11)	1.73 (1.32–2.28)
Newly OAT initiatives	4.70 (4.03–5.48)	4.73 (3.99–5.60)

HR: hazard ratio; CI: confidence interval

^a^ Variables collected on the lagged visit of treatment discontinuation outcome and refer to the last six months prior to the interview date

**Table 3:** Marginal structural Cox regression model for the effect of substance use classes on risk of OAT/methadone discontinuation among participants receiving OAT in Vancouver, Canada

	Marginal Structural Cox Model	
	aHR (95% CI)^b^	aHR (95% CI)^b^
	OAT discontinuation (N = 13,336)	Methadone discontinuation (N = 12,513)
Substance Use Class^a^		
Primarily crack use	Ref	Ref
Primarily cannabis & crack use	0.72 (0.57–0.92)	0.70 (0.54–0.90)
Primarily opioid & crack/cocaine use	1.56 (1.28–1.90)	1.57 (1.29–1.93)
Primarily opioid & meth use	1.75 (1.39–2.20)	1.66 (1.29–2.13)

aHR: adjusted hazard ratio; CI: confidence interval

^a^ Substance use classes were constructed using substance use indicators collected on the lagged visit of treatment discontinuation outcome and refer to the last six months prior to the interview date

^b^ Adjusted for age, gender, ethnicity/ancestry, cohort, unstable housing, Downtown Eastside residence, sex work involvement, incarceration, drug-dealing involvement, difficulty accessing addiction treatment, and new initiates of OAT.

## Discussion

Among individuals receiving OAT in two prospective cohorts in Vancouver, Canada, we identified four distinct longitudinal substance use latent classes and found differential OAT discontinuation risks across these classes. The latent classes representing regular use of both unregulated opioids and stimulants were associated with a higher OAT discontinuation risk. Meanwhile, of those characterized by moderate crack use in the absence of unregulated opioid use, the class representing primarily cannabis use was associated with reduced OAT discontinuation risk.

Our study noted that regular stimulant use was highly prevalent among people receiving OAT. While methamphetamine and crack/cocaine were often used with unregulated opioids, crack use was also moderately prevalent in the classes unlikely to use unregulated opioids. In addition, we observed that the increase in the co-use pattern of opioids and stimulants was primarily driven by the surging co-use pattern of unregulated opioids and methamphetamine. At the end of our study period in early 2020, nearly half of our participants receiving OAT reported regularly using both unregulated opioids and stimulants including methamphetamine and crack/cocaine; among those, two-third reported regularly using unregulated opioids and methamphetamine. This observation aligns with previous research reporting on a pattern of rising methamphetamine use among people who use opioids in several Canadian and US settings (Ellis et al., 2018; Fischer et al., 2021; Strickland et al., 2019). Compared to crack or cocaine, methamphetamine is easier to produce and thus is more widely available and has a less expense (Buxton & Dove, 2008; Farrell et al., 2019). Methamphetamine also produces higher levels of dopamine with a longer duration of effect than crack or powder cocaine (Farrell et al., 2019). With synthesized drugs rapidly contaminating the unregulated drug supply, patients could use methamphetamine to cope with the effects of more potent synthetic opioids (i.e., fentanyl) as a means to prevent over-sedation (BC Coroners Service, 2020; Ellis et al., 2018). Also, the combined use of opioids and stimulants could lead to enhanced euphoric effects and increased consumption, resulting in elevated adverse effects including respiratory depression, myocardial infarction, and overdoses (Crummy et al., 2020; Jones et al., 2012).

Our study also found that polysubstance use involving opioids and stimulants was associated with increased OAT discontinuation risk, and the relationship between the co-use of opioids and stimulants and OAT discontinuation risk was found not to be different across stimulant types (i.e., methamphetamine versus crack/cocaine). Previous research has shown stimulant use was associated with reduced OAT retention (Banta-Green et al., 2009; Deck & Carlson, 2005; Montalvo et al., 2019; Peles et al., 2008, 2018; Proctor et al., 2015; Sullivan et al., 2010; Wan Shakira et al., 2017; Williamson et al., 2006). Our findings emphasized that the co-use pattern of opioids and stimulants (especially methamphetamine) may contribute to the challenges to stabilize patients on OAT, increase the risk of treatment discontinuation and exacerbate the risk of overdose as a consequence. More intensified integrated strategies would be essential to meet the needs of these patients receiving OAT. In the current landscape of opioid use characterized by the widespread prevalence of synthetic opioids, clinicians should routinely assess their patients’ treatment satisfaction and make necessary adjustments to treatment modality and dosing. In the absence of effective pharmacological treatments for stimulant use disorders (Morley et al., 2017), care providers could consider combining OAT medications with non-pharmacological interventions (e.g., contingency management) as a strategy (Brown & DeFulio, 2020). However, it should be noted that these interventions have not been found to be durable in the long term (Morley et al., 2017). Rather than solely focusing on opioid use, future investments are urgently needed in the development of effective pharmacological treatments targeting concurrent opioid and stimulant use.

Furthermore, among the two classes characterized by moderate level of regular crack use in the absence of regular unregulated opioid use, our study showed that regular cannabis use was associated with lower rates of OAT discontinuation. While it has been consistently reported that crack cocaine use is associated with reduced OAT retention (O’Connor et al., 2020), the evidence from previous studies studying the relationship between cannabis use and OAT discontinuation has been mixed (Franklyn et al., 2017; Hser et al., 2014; Hurd et al., 2015; Scavone et al., 2013; Socías et al., 2018; Weizman et al., 2004; Zielinski et al., 2016). A retrospective chart-review analysis conducted in Philadelphia in 2013 noted some potential benefits of cannabis use in helping with opioid withdraw symptoms among patients undergoing methadone stabilization (Scavone et al., 2013). Additionally, recent research conducted in Vancouver suggested that cannabis use may help mitigate some of the negative effects of sub-therapeutic methadone dosing and help improve treatment retention (Lake et al., 2023; Socías et al., 2018). Other studies showed conflicting results. A nine-site phase IV study conducted in the US in 2014 and a retrospective cohort study conducted in Ontario, Canada in 2017 both reported cannabis use was associated with an increased risk of OAT discontinuation (Franklyn et al., 2017; Hser et al., 2014). However, neither of these two studies accounted for patients’ ongoing opioid or stimulant use status (Franklyn et al., 2017; Hser et al., 2014). Our study may help explain the contrasting findings by noting that the association between regular cannabis use and reduced risk of OAT discontinuation corresponds to a moderate likelihood of crack use and low likelihood of unregulated opioid use. In a systematic review published in 2021, Daldegan-Bueno *et al.* synthesized findings from qualitative studies and indicated that that people may use cannabis to alleviate undesirable effects of crack use (Daldegan-Bueno et al., 2021). Compared to individuals who use cannabis to counter the potential overexcitement provided by stimulants, people use only stimulants may be more inclined to turn to unregulated opioids for their sedating effects, and in doing so potentially compromise their retention on OAT. Collectively, our finding may point to a possible harm reduction strategy by substituting unregulated opioids with cannabis among people receiving OAT. The legalization of non-medical cannabis use in Canada in 2018 may offer an unprecedented opportunity to explore its potential to serve as a safer alternative to unregulated opioids (Hill & George, 2019). It may also be worthwhile to investigate the potential of medical cannabis as an adjunct treatment for OAT.

Our study has several strengths. First, our study was conducted using two ongoing prospective cohorts with over ten thousand observations collected over an extended period of time (nearly 15 years) in Vancouver, Canada. The long-standing drug use situation in Vancouver makes it an ideal setting to observe and study the complex substance use patterns continuously evolving over time (Friedel & Staak, 1993). Second, building upon the extensive data collection, we applied a person-centred analytical approach (i.e., RMLCA) and successfully identified four distinct substance use patterns among individuals receiving OAT. RMLCA has been recognized as a useful analytical strategy for describing social and behavioral phenomena (Collins LM and Lanza ST 2010). The application of such an approach made it possible to overcome the difficulties to define polysubstance use and understand the relationship between dominant substance use patterns and OAT retention. Third, we applied marginal structural modeling to account for time-varying exposure and time-varying confounders as well as censoring throughout study follow-up (Hernán et al., 2000). This approach approximates a per-protocol analysis of the target clinical trial (Hernán et al., 2000).

We also acknowledge the limitations of our study. First, the participants in both VIDUS and ACCESS were recruited with non-random sampling, therefore, the generalizability to people receiving OAT in BC or other settings may be limited. Second, data collected in this study was self-reported and therefore could be subject to recall bias and social desirability bias. However, prior research has suggested that self-reported data was generally accurate among drug-using populations (Darke, 1998). Third, we could not account for OAT adherence due to data limitations, and thus the time on OAT could be overcounted. However, our sample could reflect the complex nature of OAT engagement in practice. Fourth, the vast majority of our participants receiving OAT were on methadone, and therefore we could not separately assess all treatment modalities due to the small sample size of participants receiving OAT other than methadone. Fifth, tobacco use frequency was not collected consistently until June 2016 and was thus not included as an indicator in our analysis. We also did not include substance use variables measuring use of hallucinogen (e.g., phencyclidine), ecstasy (e.g., 3,4-methylenedioxymethamphetamine), or benzodiazepine due to their low frequencies of reported use. Moreover, we could not measure simultaneous use of drugs or the order of using drugs. Future studies could provide a more comprehensive picture of polysubstance use patterns if they make efforts to measure concurrent use and incorporate more complete substance use indicators. Last, informed by our focus on regular substance use, we used at-least-weekly substance use indicators combining the form of injection and non-injection. In the preliminary RMLCA analyses conducted using substance use frequency as separate indicators, 3247 combinations of substance use type and frequency were observed. The heterogeneities in substance use patterns made it challenging to identify analytically or clinically meaningful substance use classes (results not shown). Although our grouping may aid interpretability and simplify related clinical implications, it is crucial to acknowledge that complex substance use patterns may be somewhat oversimplified, potentially leading to the omission of essential nuances between individuals. Our results shed light on the complexity of possible substance use patterns and emphasize the significance of adopting a person-centered approach. To optimize treatment satisfaction and retention, it is crucial to customize treatment and social support referrals to address the specific needs of each patient.

In conclusion, our study emphasized the heterogeneities in substance use patterns among patients receiving OAT. Novel treatment strategies targeting individuals’ polysubstance use are essential to improve OAT retention and success. In particular, methamphetamine was increasingly commonly used with unregulated opioids among patients receiving OAT. Integrated treatment strategies are in urgent need to cope with the rapidly rising methamphetamine-opioid use patterns. Also, future research should be conducted to further explore the cannabis-opioid interacting effect on OAT retention and explore the therapeutic potential of cannabis as an adjunctive treatment to help improve OAT retention.

## Electronic supplementary material

Below is the link to the electronic supplementary material.

Additional File 1: Figure S1. Three latent substance use class item response probability loadings on substance use indicators among participants receiving OAT in Vancouver, Canada
