# Supplementary material for: The Impact of Longitudinal Substance Use Patterns on the Risk of Opioid Agonist Therapy Discontinuation: A Repeated Measures Latent Class Analysis
Source: Int J Ment Health Addict. 2023 Jun 28;22(6):4004–20. doi: 10.1007/s11469-023-01098-8 (PMC11666779; doi:10.1007/s11469-023-01098-8)
Supplement: Supplementary file 1 — Additional File 1: Figure S1. Three latent substance use class item response probability loadings on substance use indicators among participants receiving OAT in Vancouver, Canada [file 11469_2023_1098_MOESM1_ESM.docx]

Figure S1. Three latent substance use class item response probability loadings on substance use indicators among participants receiving OAT in Vancouver, Canada

*the three-class model was chosen due to its best interpretability, utility and parsimony of the classes suggested by the lowest BIC and CAIC value
